# Clinical characteristics and outcomes of influenza A and B virus infection in adult Australian hospitalised patients

**DOI:** 10.1186/s12879-020-05670-8

**Published:** 2020-12-01

**Authors:** Yogesh Sharma, Chris Horwood, Paul Hakendorf, Campbell Thompson

**Affiliations:** 1 College of Medicine and Public Health, Flinders University, Adelaide, SA Australia; 2 Department of General Medicine, Division of Medicine, Cardiac & Critical Care, Flinders Medical Centre, Flinders Drive, Bedford Park, Adelaide, SA 5042 Australia; 3 Department of Clinical Epidemiology, Flinders Medical Centre, Adelaide, SA Australia; 4 Discipline of Medicine, The University of Adelaide, Adelaide, SA Australia

**Keywords:** Influenza, Complications of influenza, Influenza related hospitalisation, Influenza and mortality, Influenza and readmissions

## Abstract

**Background:**

Influenza B is often perceived as a less severe strain of influenza. The epidemiology and clinical outcomes of influenza B have been less thoroughly investigated in hospitalised patients. The aims of this study were to describe clinical differences and outcomes between influenza A and B patients admitted over a period of 4 years.

**Methods:**

We retrospectively collected data of all laboratory confirmed influenza patients ≥18 years at two tertiary hospitals in South Australia. Patients were confirmed as influenza positive if they had a positive polymerase-chain-reaction (PCR) test of a respiratory specimen. Complications during hospitalisation along with inpatient mortality were compared between influenza A and B. In addition, 30 day mortality and readmissions were compared. Logistic regression model compared outcomes after adjustment for age, Charlson index, sex and creatinine levels.

**Results:**

Between January 2016–March 2020, 1846 patients, mean age 66.5 years, were hospitalised for influenza. Of whom, 1630 (88.3%) had influenza A and 216 (11.7%) influenza B. Influenza B patients were significantly younger than influenza A. Influenza A patients were more likely be smokers with a history of chronic obstructive pulmonary disease (COPD) and ischaemic heart disease (IHD) than influenza B. Complications, including pneumonia and acute coronary syndrome (ACS) were similar between two groups, however, septic shock was more common in patients with influenza B. Adjusted analyses showed similar median length of hospital stay (LOS), in hospital mortality, 30-day mortality and readmissions between the two groups.

**Conclusions:**

Influenza B is less prevalent and occurs mostly in younger hospitalised patients than influenza A. Both strains contribute equally to hospitalisation burden and complications.

**Trial registration:**

Australia and New Zealand Clinical Trial Registry (ANZCR) no ACTRN12618000451202 date of registration 28/03/2018

## Background

Influenza is a major cause of hospitalisation during winter season and is associated with significant morbidity and mortality not only due to the direct effects of viral illness but also due to complications arising because of exacerbation of chronic medical conditions. Worldwide, influenza leads to more than a million deaths annually while in Australia there were 29,000 hospitalisations and 745 deaths were attributed to influenza in 2017 [1–3].

Although influenza A is the most common type of viral infection, in 2015 influenza B (predominantly B/Yamagata) accounted for 62% of all influenza related notifications in Australia [4]. To date, influenza B has received less attention than influenza A, this is partly because influenza B is often regarded as less virulent and hence less likely to cause a more severe illness than influenza A and also because influenza A has the capacity to cause epidemics [5]. Nonetheless, its clinical importance is increasingly been recognised along with the need to characterise differences with the other influenza strains because influenza B shares significant influenza burden annually. In addition, accurate identification of influenza types is gaining importance because antiviral resistance is increasingly becoming more prevalent among influenza subtypes [6]. This is especially important in settings with a complex pattern of antiviral resistance, where prompt identification of influenza types and subtypes will be especially useful to guide antiviral treatment.

Although several studies [7–9] have described patient characteristics and outcomes with influenza A (HINIpdm09), limited studies have compared whether these characteristics/outcomes are any different from those who have influenza B infection. Moreover, only few studies [5, 10] have compared characteristics and outcomes between influenza A and B among patients who need hospitalisation. These studies are further limited by small sample sizes and single site experiences. A recent study [11] compared characteristics and outcomes between influenza types/subtypes enrolled only adult patients and has suggested the need for ongoing research to assess changing severity patterns among types/subtypes over time. Therefore, the aims of this study were to describe patient characteristics and compare clinical outcomes among hospitalised patients with influenza A and B.

## Methods

This study was designed as a retrospective observational study and included all patients ≥18 years, admitted with a diagnosis of influenza, at the two major tertiary hospitals of South Australia between January 2016 and April 2020. The Royal Adelaide Hospital (RAH) is an 800 bed teaching hospital that provides care to 85,000 inpatients each year and caters to 461,000 residents of central Adelaide. Flinders Medical Centre (FMC) is a 590 bed teaching hospital which provides services to the southern metropolitan area of Adelaide with a population of 371,000. We included patients who were ≥ 18 years because only limited studies have compared influenza A and B in adults in the Australian health care settings. Influenza related hospitalisations were identified by the International Statistical Classification of Diseases and Related Health Problems, 10th Revision, Australian Modification (ICD-10-AM) [12] diagnosis code of influenza (ICD-10-AM J9 or J10) and a laboratory confirmed influenza infection. Eligible cases for this study required confirmation of influenza through collection of nasal/oropharyngeal swabs for influenza A and B and analysed by the reverse transcription polymerase chain reaction (RT-PCR) test [13]. Ethical approval was granted by the Southern Adelaide Human Clinical Research Ethics Committee (SA HREC) and this study was registered with the Australia and New Zealand Clinical Trial Registry (ANZCR) no 12618000451202.

### Data collection

From the electronic medical records, we abstracted the following variables for all patients who had had a hospital admission with laboratory confirmed influenza infection: age, sex, Charlson Comorbidity Index (CCI) [14], smoking status, history of diabetes, IHD, COPD, bronchial asthma, interstitial lung disease (ILD), chronic kidney disease (CKD), malignancy and immunosuppression. We recorded creatinine, c-reactive protein (C-RP) and high sensitive troponins (hsTnT) available during hospital admission. We also recorded various complications arising during the course of hospital admission: pneumonia, respiratory failure, acute respiratory distress syndrome (ARDS), ACS and sepsis (defined as a life threatening organ dysfunction caused by a suspected infection and associated with any 2 of the following criteria: Glasgow Coma Scale < 13, systolic blood pressure < 100 mmHg, and respiratory rate > 22/min. Septic shock was defined as sepsis and need for vasopressor support to maintain blood pressure and hyperlactataemia. The severity of influenza related illness was described by the number of medical emergency response team (MET) calls, the proportion of patients who had multiple (≥ 2) MET calls during admission, the proportion of patients who went to the intensive care unit (ICU), time spent in hours in the ICU and inpatient mortality. The LOS was adjusted for inpatient mortality. We also recorded mortality and unplanned hospital readmissions within 30 days following discharge.

### Statistics

Data were assessed for normality by visual inspection of histograms. We compared the clinical characteristics of patients and outcomes between the influenza types by use of chi squared statistics or Fisher’s exact test for categorical variables and student’s t-test or rank-sum test were used for continuous variables, where appropriate. Analysis of variance (ANOVA) compared rates of complications such as time spent in the ICU and outcomes (LOS) over a period of 4 years form 2016–2020 to assess virulence of influenza viruses in different seasons. Any significant differences in complications between the two influenza types were adjusted for age, sex, CCI, creatinine and diabetes status of the patients. We used logistic regression to determine the odds ratios for mortality outcomes between the two influenza types after adjustment for confounders: age, sex, CCI and creatinine levels. We compared mortality and unplanned hospital readmissions at 30 days following hospital discharge by use of survival curves and log-rank test compared survival differences between the two influenza types. All tests were 2 sided and a *P* value of < 0.05 was regarded as statistically significant. All statistical analyses were conducted using STATA software version 16 (STATA corp., Texas).

## Results

**Fig. 1:**
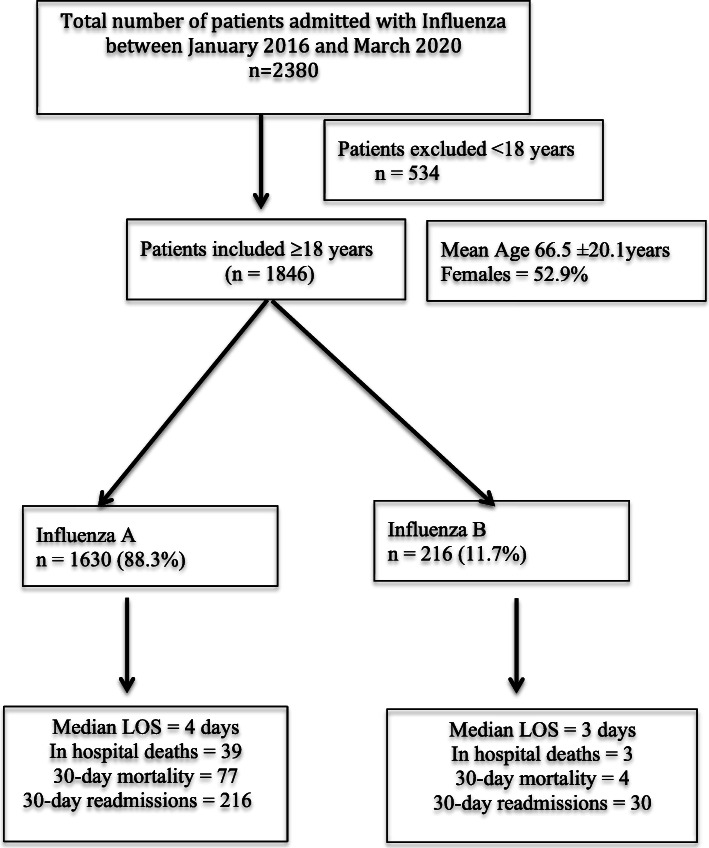
Study flow diagram

Between January 2016 to March 2020, 2380 hospitalised patients tested positive for influenza, of whom, 1846 were ≥ 18 years of age and were included in this study (Fig. 1). One thousand six hundred and thirty (88.3%) patients had influenza A and 216 (11.7%) influenza B. The annual prevalence of influenza A vs influenza B varied between 74.9 to 95.1% and of influenza B from 4.9 to 25.1% in seasons between 2016 and 2019. Highest prevalence (95.1%) of Influenza A was recorded in 2016 and of influenza B (25.1%) in 2018. The mean age was 66.5 ± 20.1 years (range 18–101) and 52.9% were females. Patients with influenza B were significantly younger than influenza A. Forty six (21.3%) influenza B patients were younger than 40 years, while 555 (34.1%) influenza A patients were older than 80 years of age (Table 1). There was no difference in sex or the number of comorbidities, as determined by the CCI between the two groups. However, patients who had influenza A were more likely to be smokers, to have a history of IHD or of COPD than those who had influenza B infection and these differences might be related to the older age of participants with influenza A. Other characteristics were similar between the two groups (Table 1). The C-RP levels were significantly higher among patients with influenza A patients (*P* <  0.001) but creatinine and hsTnT levels were similar between the two groups. A similar number of influenza A and B patients had hsTnT performed during admission (40.7% vs. 39.6%, *P* value = 0.796) and hsTNT was positive in similar number of patients (41.8% vs. 31.4%, *P* value = 0.063). Among patients who had a positive hsTnT, the diagnosis of ACS was not significantly different between the two groups (48 (17.5%) vs. 3 (11.1%), *P* value = 0.401). Overall, 448 (24.5%) patients were treated with Oseltamivir, with significantly higher number of influenza A patients receiving antiviral treatment as compared to influenza B (25.7% vs. 15.6%, *P* = 0.001) (Table 1).

**Table 1:** Patient characteristics

Variable	Influenza A	Influenza B	***P*** value
	*N* = 1630	*N* = 216	
Age (years) mean (SD)	67.3 (19.9)	60.1 (20.5)	< 0.001
Age group n (%)			< 0.001
< 40	219 (13.4)	46 (21.3)	
40–59	261 (16.0)	49 (22.7)	
60–79	595 (36.5)	79 (36.6)	
> 80	555 (34.1)	42 (19.4)	
Females n (%)	827 (52.6)	102 (55.4)	0.473
CCI mean (SD)	1.30 (1.61)	1.33 (1.85)	0.648
Smokers n (%)	779 (47.8)	87 (40.3)	< 0.038
Diabetes n (%)	407 (24.9)	45 (20.8)	0.0184
COPD n (%)	447 (27.4)	44 (20.4)	< 0.027
Asthma n (%)	110 (6.8)	15 (6.9)	0.914
ILD n (%)	16 (0.8)	4 (1.9)	0.246
CKD n (%)	17 (1.0)	1 (0.4)	0.415
IHD n (%)	307 (18.8)	24 (11.1)	< 0.005
Malignancy n (%)	181 (11.1)	28 (12.9)	0.418
Immunosuppression n (%)	7 (0.4)	3 (1.4)	0.071
^a^hsTnT mean (SD)	76.6 (379.5)	55.9 (88.4)	0.614
^b^Creatinine mean (SD)	107.8 (105.8)	103.9 (114.1)	0.626
^c^C-RP mean (SD)	60. 2 (71.9)	42.9 (64.2)	0.002
Antiviral treatment n (%)	415 (25.7)	33 (15.6)	0.001

*CCI* Charlson comorbidity index, *COPD* Chronic obstructive pulmonary disease, *ILD* Interstitial lung disease, *CKD* Chronic kidney disease, *IHD* Ischaemic heart, *hsTnT* High sensitive troponin, *C-RP* C-reactive protein

^a^ng/L

^b^micromol/L

^c^mg/L

When compared to patients who had influenza A, influenza B patients had similar risk of developing pneumonia or respiratory failure. The severity of illness, as determined by the number of MET calls, proportion of patients who had multiple (≥ 2 MET calls) during admission (3.3% vs. 0.7%, *P* = 0.229), time spent in the ICU and the proportion of patients who were admitted in the ICU (7.6% vs. 8.8%, *P* = 0.517) was also similar between the two groups (Table 2). However, a higher proportion of patients who had influenza B developed septic shock (3.8% vs. 0.5%, *P* <  0.001) when compared to those who had influenza A (Table 2). This risk remained significant after adjustment for confounders such as age, CCI, diabetes and creatinine levels (OR 7.62, 95% CI 2.55 to 23.10, *P* <  0.001).

**Table 2:** Complications and outcomes between influenza A and B

Variable	Influenza A	Influenza B	***P*** value
	*n* = 1630	*n* = 216	
^a^LOS median (iqr)	4 (5)	3 (5)	< 0.435
Inpatient mortality	39 (2.4)	3 (1.8)	< 0.353
30-day mortality	77 (4.7)	4 (1.2)	0.041
30-day readmissions	216 (13.3)	30 (13.9)	0.796
Pneumonia	31 (11.2)	1 (3.1)	0.408
Respiratory failure	40 (14.5)	6 (22.2)	0.289
ARDS	15 (0.9)	0	0.183
ACS	50 (3.2)	3 (1.6)	0.244
MET calls mean (SD)	0.15 (0.57)	0.08 (0.29)	0.138
Patients with MET calls n (%)	93 (9.4)	10 (7.0)	0.364
Patients with ≥2 MET calls n (%)	33 (3.3)	1 (0.7)	0.229
ICU hours	13.9 (85.2)	13.9 (67.4)	0.990
Patients needing ICU admission n (%)	123 (7.6)	19 (8.8)	0.517
Sepsis	70 (4.5)	16 (8.7)	0.012
Septic shock	7 (0.5)	7 (3.8)	< 0.001
Myocarditis	1 (0.1)	1 (0.5)	0.068
Pericarditis	1 (0.1)	0	0.732

*LOS* Length of hospital stay, *iqr* Interquartile range, *ARDS* Adult respiratory distress syndrome, *ACS* Acute coronary syndrome, *MET* Medical emergency response team, *ICU* Intensive care unit

^a^LOS adjusted for inpatient mortality

**Fig. 2:**
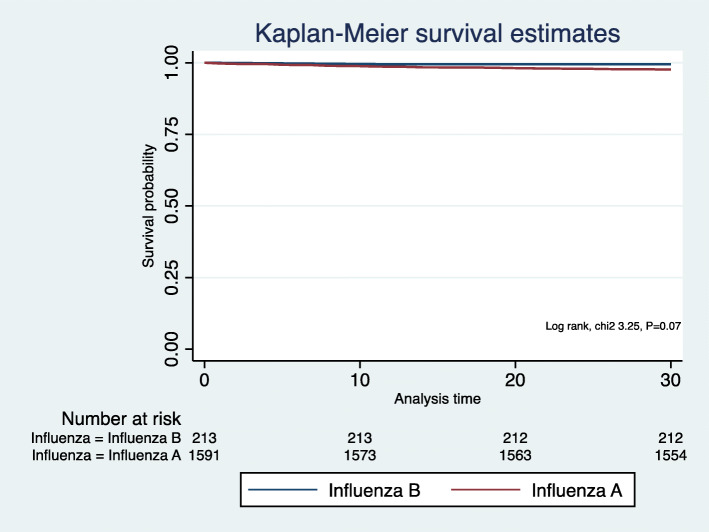
Kaplan-Meier survival curve

Median LOS was similar between influenza A and B (median LOS 4 (IQR 5) days vs. 3 (IQR 4) days, *P* = 0.435). Inpatient mortality was also similar between the two groups (Table 2). At 30 days following hospital discharge, 77 (4.7%) patients who had influenza A died as compared to 4 (1.2%) with influenza B (*P* <  0.041). However, logistic regression model showed that mortality at 30 days of hospital discharge was not higher in patients with influenza A after adjustment for age, sex, CCI and creatinine (aOR 3.61, 95% CI 0.43 to 30.01, *P* = 0.235) when compared to influenza B. Kaplan-Meier survival curve (Fig. 2) showed no survival difference between the two groups within 30 days of hospital discharge (log-rank test chi2 3.25, *P* = 0.07). There was no difference in the number of 30-day unplanned hospital readmissions between the two groups (*P* value < 0.05) (Table 2).

Clinical outcomes, such as LOS, in-hospital mortality, 30-day mortality and readmissions in individual years from 2016 to 2019 were also similar (*P* > 0.05) between the two influenza types.

## Discussion

This study suggests that over a period of 4 years, influenza A was associated with a greater number of hospital admissions as compared to influenza B. This is indicative of a greater prevalence of circulating influenza A viruses circulating in the community during the seasons in the study period, as has also been suggested by previous studies [15, 16]. Influenza B infection, however, was more prevalent in younger population but clinical outcomes measured in terms of LOS, ICU admissions, in-hospital mortality, 30-day mortality and unplanned hospital readmissions were comparable to influenza A.

The results of our study are similar to a study by Cohen et al. [17] in Israel, who included 454 hospitalised influenza patients with a mean age 66 years over a period of 3 years and found minor epidemiological differences but similar clinical severity and outcomes between influenza A and B viral infections. However, the prevalence of influenza B infection was much higher 32.5% in their study as compared to 11.7% in our study. The prevalence rates of different influenza strains in our study are, however, similar to a report by the influenza complications alert network (FluCAN) [15], which reports annual flu data in 22 sentinel hospitals across Australia, and in 2018 found that among 769 hospitalised patients, influenza B constituted 13% of the total influenza burden. A meta-analysis [18] of studies conducted in Latin America and the Caribbean, between 1999 to 2008 suggests that overall influenza A is more common than influenza B, although there were a few years in a few countries where there was a high percentage of influenza B positive cases than influenza A. The differences in the prevalence of flu strains could be related to varying circulation of the two influenza viruses during different seasons [19]. In our study, we found that the prevalence of influenza B among hospitalised patients has varied from 4.9 to 25.1% from 2016 to 2019.

The prevalence rate of influenza B in our study is lower than another Australian study [20], which studied epidemiology of all laboratory confirmed influenza B infections for inpatients and outpatients across Australia between 2001 and 2014 influenza seasons and found that the prevalence of influenza B was 17.1% compared to 11.7% in our study. This slight difference in reporting might be related to the sampling characteristics, because our study included only hospitalised patients compared to inclusion of all influenza cases in their study. In addition, these differences could be related to the under representation of influenza B among hospitalised patients, because, influenza B is more common in younger patients who often have fewer co-morbidities and thus are less likely to present to hospital for exacerbation of chronic medical issues. The other reason for difference in prevalence rates of influenza viruses could be related to the seasonal variation in circulating influenza strains, as our reporting period was from 2016 to 2020 compared to 2001–2014 in the other study. In addition, it is possible that hospitalisation for flu is determined by the co-morbidities rather than the type of virus with people who are older with multiple comorbidities more likely to be admitted than younger patients with no or fewer medical issues.

The mean age of our study population was 66.5 years which is much older than a previous Australian study [21] in 2015, which included 436 adult hospitalised influenza patients who had a mean age of 54.3 years. This difference in age is likely related to the higher prevalence of influenza B (59.3%) in this other study compared to our population (11.7%). Influenza B is more prevalent in younger population and was the dominant strain in Australia in 2015. Similar to previous studies [20, 22], our study also confirms that influenza B is more prevalent in young adults while influenza A is more common in older hospitalised patients. It is unclear why influenza B is more prevalent in younger adults and children as compared to influenza A. The increased susceptibility of younger population might be related to certain viral and host factors, however, these have not been determined yet and needs further research.

In this study, the patient characteristics and background morbidity were similar between influenza A and B, apart from differences in smoking status and history of IHD, which were significantly higher among patients who had influenza A. These findings are similar to a recent study by Cohen et al. [17], who included 759 hospitalised influenza patients and found only minor epidemiological differences between the two influenza types but noted a higher prevalence of IHD among influenza A patients. Despite high prevalence of IHD, we did not observe any significant differences in the incidence of ACS (3.2% vs. 1.6%, *P* = 0.244) or finding of an elevated hsTnT levels (41.8% vs. 31.4, *P* value 0.063) between the two influenza types. Moreover, similar to a previous study [17] both myocarditis and pericarditis were rare complications of influenza and were not significantly different between the two types of influenza. In addition, these complications remained similar between the two groups in individual years between 2016 and 2019. These finding suggest that both influenza strains A and B are equally associated with cardiac complications.

Pneumonia is a major complication of influenza that may be associated with mortality, other common complications arise due to exacerbation of chronic medical issues, while cardiac and neurological complications e.g. encephalitis are rare [23, 24]. Known risk factors for complications of influenza include: older age, preexisting cardiopulmonary disease, immunosuppression, obesity, neurological and other common medical conditions [25, 26]. In our study, complications were common and pneumonia developed in 7.2% of influenza patients, of whom, 18.9% developed respiratory failure, however, we could not find any significant difference in clinical outcomes among patients who developed complications between the two influenza types. Cohen et al. [17] in their study in hospitalised influenza patients, found that 19% of influenza patients developed pneumonia but there were no differences in outcomes between influenza A and B. The higher rate of pneumonia in their study was likely related to the higher prevalence of multi-morbidity in their patients as reflected by the higher mean CCI (4.3 vs. 1.3) compared to our population.

In our study, a significantly higher proportion of patients who had influenza B developed septic shock as compared to those who had influenza A (3.8% vs. 0.5%, *P* <  0.001). Patients with influenza B, who developed septic shock were more likely to be younger than 60 years and had a diagnosis of pneumonia and a lower CCI than influenza A. Moreover, there were no significant differences with respect to receiving Oseltamivir or antibiotics between the two groups (*P* > 0.05). This indicates that influenza B in some cases may cause a severe illness in previously well individuals. Previous literature has not looked into this clinical outcome, however, case reports [26, 27] suggest that influenza B can pose a risk of severe secondary infection in previously healthy individuals.

We found no significant differences in clinical outcomes such as LOS, inpatient mortality and mortality and readmissions within 30 days following hospital discharge among patients with influenza A and B. In addition, the annual rates of these outcomes over the four influenza seasons, between 2016 and 2020, were similar between influenza A and B (*P* > 0.05), indicating similar virulence pattern of the two influenza types during this period. These results are similar to previous studies [17, 28] who also found no difference in LOS and in hospital mortality between influenza A and B viral infections, however, data on long term clinical outcomes after hospitalisation for influenza are scarce. These results indicate that contrary to some of the previous studies [28, 29] influenza B cannot be regarded as a milder infection as compared to influenza A especially among hospitalised patients.

In terms of clinical outcomes such as LOS and mortality, our results are similar to previous studies conducted even in hospitalised paediatric populations which also showed no significant differences in these outcomes between influenza A and B patients [10, 30]. It is potentially concerning that younger patients with influenza B who needed hospitalisation, despite having lesser comorbidity had similar clinical outcomes, when compared to influenza A and thus demands consideration for an early antiviral therapy. Some studies suggest that outpatients with influenza A (H3N2) sought care earlier than influenza B and influenza related mortality was highest during influenza A (H3N2) predominant seasons [6, 31]. Our study has limitations as we did not have information about influenza A subtypes thus stratification of analysis according to influenza A subtypes could not be performed.

The use of anti-viral treatment in our study was only 24.5% and significantly more patients who had influenza A received antivirals than influenza B. These results are significantly lower than that reported by a U.S. study [32], who found that 80% hospitalised adult influenza patients had received anti-viral treatment. These differences could be related to the lack of diagnostic confirmation of influenza during the recommended period for anti-viral treatment recommendation (i.e. within 48 h of onset of symptoms) [33]. The reason for reduced prescription of Oseltamivir in influenza B could be related to the fact that influenza B patients, being relatively younger and with fewer co-morbidities, were perceived by their clinicians to be at a relatively reduced risk of developing complications than influenza A patients. In addition, as previous studies [34, 35] have shown a decreased efficacy of Oseltamivir in influenza B when compared to influenza A, this could have discouraged clinicians in prescribing antivirals to influenza B patients. However, similar to other studies [36, 37] we also found that hospitalised influenza patients, who received antiviral treatment were more likely to have a less severe illness as reflected by fewer ICU admissions (19.8% vs. 24.9%) and significantly lower in hospital mortality (1.1% vs. 2.5%, *P* <  0.05) than those who did not receive anti-viral therapy.

This study has some limitations. Information about vaccination status of the participants was unavailable. Evidence [38], suggests that influenza vaccination reduces cardiovascular events and mortality and it is possible that vaccination may have impacted mortality in this study as annual influenza vaccination coverage in adults in Australia is 54% [39].

## Conclusions

The findings of this study suggest that influenza B infection although less prevalent than influenza A, causes similar morbidity among hospitalised patients and thus should not be regarded as a less severe infection.

## Data Availability

The datasets used and analysed in the current study are available from the corresponding author on reasonable request.

## Abbreviations

ACS: Acute coronary syndrome
ANOVA: Analysis of variance
ARDS: Acute respiratory distress syndrome
CCI: Charlson comorbidity index
CI: Confidence interval
CKD: Chronic kidney disease
COPD: Chronic obstructive pulmonary disease
C-RP: C-reactive protein
FluCAN: Influenza complications alert network
hsTnT: High sensitive troponins
ICD-10-AM: International Statistical Classification of Diseases and Related Health Problems, 10th Revision, Australian Modification
ICU: Intensive care unit
IHD: Ischaemic heart disease
ILD: Interstitial lung disease
LOS: Length of hospital stay
MET: Medical emergency response team
OR: Odds ratio
PCR: Polymerase chain reaction
